# Supplementary material for: Associations between Long-Term Air Pollution Exposure and Risk of Osteoporosis-Related Fracture in a Nationwide Cohort Study in South Korea
Source: Int J Environ Res Public Health. 2022 Feb 19;19(4):2404. doi: 10.3390/ijerph19042404 (PMC8872590; doi:10.3390/ijerph19042404)
Supplement: Supplementary file 1 [file ijerph-19-02404-s001.zip › ijerph-1547173-supplementary.pdf]

Table S1. Descriptive statistics of included and excluded participants of the NHIS-NCS data (2002-2015).

	**Excluded participants (n = 28,046)**	**Included participants (n = 56,498)**	***p*-value**
Sex (n, %)			
Men	12,856 (45.8)	26,258 (46.5)	0.080
Women	15,190 (54.2)	30,240 (53.5)	
Age at the entry year (n, %)			
50-59y	5,001 (17.8)	20,683 (36.6)	<0.001
60-64y	4,526 (16.1)	15,191 (26.9)	
65-69y	5,558 (19.8)	11,517 (20.4)	
70-74y	5,126 (18.3)	5,854 (10.4)	
75-79y	4,319 (15.4)	2,451 (4.3)	
80-84y	3,033 (10.8)	721 (1.3)	
85-89y	483 (1.7)	81 (0.1)	
Insurance fee (n, %)			
0-19th percentiles	2,224 (8.7)	4,653 (8.4)	0.160
20-39th percentiles	3,556 (14.0)	7,463 (13.5)	
40-59th percentiles	4,134 (16.2)	9,180 (16.6)	
60-79th percentiles	4,582 (18.0)	9,924 (18.0)	
80-100th percentiles	10,978 (43.1)	24,007 (43.5)	

Table S2. Pearson correlation coefficients of annual mean concentration (2002-2013) among air pollutants.

	**PM_{10} ($\mu g/m^3$)**	**SO_2 (ppm)**	**CO (ppm)**	**NO_2 (ppm)**	**O_3 (ppm)**
PM_{10} ($\mu g/m^3$)	1	0.210	0.354	0.383	-0.508
SO_2 (ppm)		1	0.057	0.124	0.033
CO (ppm)			1	0.470	-0.513
NO_2 (ppm)				1	-0.691
O_3 (ppm)					1

Table S3. Cox proportional Hazard Ratios (HRs) of 3-year moving annual average of air pollution for an IQR increase in two-pollutant models for osteoporosis-related fracture in seniors (age ≥50)(n= 56,467).

**Pollutant**	**Two-pollutants model adjusted for pollutant on the left column**				
	**PM_{10}**	**SO_2**	**CO**	**NO_2**	**O_3**
PM_{10} (13.7$\mu g/m^3$)	1.00 (0.93, 1.07)	1.05 (1.00, 1.09)	0.99 (0.94, 1.04)	1.00 (0.95, 1.05)	1.00 (0.95, 1.06)
SO_2 (0.002 ppm)	0.98 (0.91, 1.05)	1.04 (1.00, 1.09)	0.98 (0.94, 1.03)	0.99 (0.94, 1.04)	1.00 (0.95, 1.06)
CO (0.192 ppm)	1.00 (0.93, 1.07)	1.04 (1.00, 1.09)	0.99 (0.95, 1.04)	1.01 (0.95, 1.06)	1.00 (0.94, 1.06)
NO_2 (0.012 ppm)	1.00 (0.93, 1.08)	1.05 (1.00, 1.09)	0.99 (0.93, 1.04)	1.01 (0.96, 1.05)	1.01 (0.93, 1.10)
O_3 (0.007 ppm)	1.00 (0.93, 1.08)	1.04 (1.00, 1.09)	0.99 (0.94, 1.04)	1.01 (0.93, 1.09)	1.00 (0.95, 1.05)

Note: HRs in diagonal cells are the results from single-pollutant models.

Table S4. Fully adjusted Cox model PM_{10} hazard ratios (and 95% CIs) per IQR increase in 3-year moving annual average PM_{10} for osteoporosis-related fracture incidence and hazard ratios of covariates (n=56,467).

	HR (95% CI)
PM_{10} (13.7 $\mu g/m^3$)	1.00 (0.93, 1.07)
Diagnosis of rheumatoid arthritis	
Yes vs. no	1.01 (0.92, 1.11)
Diagnosis of causes of secondary osteoporosis	
Yes vs. no	1.11 (1.01, 1.21)
Exposure to oral glucocorticoids	
Yes vs. no	1.28 (1.13, 1.46)
Use of anti-osteoporosis agents	
Yes vs. no	3.04 (2.81, 3.28)
CCI	1.02 (1.00, 1.03)
BMI (10 kg/m^2)	0.85 (0.77, 0.93)
Smoking status	
Never smoker	Reference
Former smoker	1.06 (0.93, 1.2)
Current smoker	1.25 (1.11, 1.4)
High alcohol intake	
Yes vs. no	1.27 (1.07, 1.52)
Frequency of exercise per week	
Never	Reference
1-2 times	0.95 (0.85, 1.07)
3-4 times	0.85 (0.73, 0.98)
5-6 times	1.02 (0.82, 1.28)
Everyday	0.96 (0.89, 1.03)
Income	
1-39th percentiles	Reference
40-79th percentiles	0.94 (0.86, 1.02)
80-100th percentiles	0.93 (0.86, 1.01)

Note: CCI = Charlson Comorbidity Index, BMI = body mass index. BMD = bone mineral density.

Table S5. Fully adjusted Cox model SO_2 hazard ratios (and 95% CIs) per IQR increase in 3-year moving annual average SO_2 for osteoporosis-related fracture incidence and hazard ratios of covariates (*n* = 56,467).

	**HR (95% CI)**
SO_2 (0.002 ppm)	1.04 (1.00, 1.09)
Diagnosis of rheumatoid arthritis	
Yes vs. no	1.01 (0.92, 1.11)
Diagnosis of causes of secondary osteoporosis	
Yes vs. no	1.11 (1.01, 1.21)
Exposure to oral glucocorticoids	
Yes vs. no	1.28 (1.13, 1.46)
Use of anti-osteoporosis agents	
Yes vs. no	3.04 (2.81, 3.28)
CCI	1.02 (1.01, 1.03)
BMI (10 kg/m^2)	0.85 (0.77, 0.93)
Smoking status	
Never smoker	Reference
Former smoker	1.06 (0.93, 1.20)
Current smoker	1.24 (1.10, 1.40)
High alcohol intake	
Yes vs. no	1.27 (1.07, 1.52)
Frequency of exercise per week	
Never	Reference
1-2 times	0.96 (0.85, 1.07)
3-4 times	0.85 (0.74, 0.99)
5-6 times	1.03 (0.82, 1.28)
Everyday	0.96 (0.89, 1.03)
Income	
1-39th percentiles	Reference
40-79th percentiles	0.94 (0.86, 1.02)
80-100th percentiles	0.93 (0.87, 1.01)

Table S6. Fully adjusted Cox model CO hazard ratios (and 95% CIs) per IQR increase in 3-year moving annual average CO for osteoporosis-related fracture incidence and hazard ratios of covariates (*n* = 56,467).

	**HR (95% CI)**
CO (0.192 ppm)	0.99 (0.94, 1.04)
Diagnosis of rheumatoid arthritis	
Yes vs. no	1.01 (0.92, 1.11)
Diagnosis of causes of secondary osteoporosis	
Yes vs. no	1.11 (1.01, 1.21)
Exposure to oral glucocorticoids	
Yes vs. no	1.28 (1.13, 1.46)
Use of anti-osteoporosis agents	
Yes vs. no	
CCI	1.02 (1.00, 1.03)
BMI (10 kg/m^2)	0.85 (0.77, 0.93)
Smoking status	
Never smoker	Reference
Former smoker	1.06 (0.93, 1.2)
Current smoker	1.24 (1.1, 1.4)
High alcohol intake	
Yes vs. no	1.27 (1.07, 1.52)
Frequency of exercise per week	
Never	Reference
1-2 times	0.95 (0.85, 1.07)
3-4 times	0.85 (0.73, 0.98)
5-6 times	1.02 (0.82, 1.28)
Everyday	0.96 (0.89, 1.03)
Income	
1-39th percentiles	Reference
40-79th percentiles	0.94 (0.86, 1.02)
80-100th percentiles	0.93 (0.86, 1.01)

Table S7. Fully adjusted Cox model NO_2 hazard ratios (and 95% CIs) per IQR increase in 3-year moving annual average NO_2 for osteoporosis-related fracture incidence and hazard ratios of covariates (*n* = 56,467).

	**HR (95% CI)**
NO_2 (0.012 ppm)	1.00 (0.96, 1.05)
Diagnosis of rheumatoid arthritis	
Yes vs. no	1.01 (0.92, 1.11)
Diagnosis of causes of secondary osteoporosis	
Yes vs. no	1.11 (1.01, 1.21)
Exposure to oral glucocorticoids	
Yes vs. no	1.28 (1.13, 1.46)
Use of anti-osteoporosis agents	3.04 (2.81, 3.28)
Yes vs. no	
CCI	1.02 (1, 1.03)
BMI (10 kg/m^2)	0.85 (0.77, 0.93)
Smoking status	
Never smoker	Reference
Former smoker	1.06 (0.93, 1.2)
Current smoker	1.25 (1.11, 1.4)
High alcohol intake	
Yes vs. no	1.27 (1.07, 1.52)
Frequency of exercise per week	
Never	Reference
1-2 times	0.95 (0.85, 1.07)
3-4 times	0.85 (0.73, 0.98)
5-6 times	1.02 (0.82, 1.28)
Everyday	0.96 (0.89, 1.03)
Income	
1-39th percentiles	Reference
40-79th percentiles	0.94 (0.86, 1.02)
80-100th percentiles	0.93 (0.86, 1.01)

Table S8. Fully adjusted Cox model O_3 hazard ratios (and 95% CIs) per IQR increase in 3-year moving annual average in O_3 for osteoporosis-related fracture incidence and hazard ratios of covariates (n = 56,467).

	**HR (95% CI)**
O_3 (0.007 ppm)	1.00 (0.95, 1.06)
Diagnosis of rheumatoid arthritis	
Yes vs. no	1.01 (0.92, 1.11)
Diagnosis of causes of secondary osteoporosis	
Yes vs. no	1.11 (1.01, 1.21)
Exposure to oral glucocorticoids	
Yes vs. no	1.28 (1.13, 1.46)
Use of anti-osteoporosis agents	
Yes vs. no	3.04 (2.81, 3.28)
CCI	1.02 (1.00, 1.03)
BMI (10 kg/m^2)	0.85 (0.77, 0.93)
Smoking status	
Never smoker	Reference
Former smoker	1.06 (0.93, 1.2)
Current smoker	1.25 (1.11, 1.4)
High alcohol intake	
Yes vs. no	1.27 (1.07, 1.52)
Frequency of exercise per week	
Never	Reference
1-2 times	0.95 (0.85, 1.07)
3-4 times	0.85 (0.73, 0.98)
5-6 times	1.02 (0.82, 1.28)
Everyday	0.96 (0.89, 1.03)
Income	
1-39th percentiles	Reference
40-79th percentiles	0.94 (0.86, 1.02)
80-100th percentiles	0.93 (0.86, 1.01)
